# A *Chrysoporthe cubensis* enzyme cocktail produced from a low-cost carbon source with high biomass hydrolysis efficiency

**DOI:** 10.1038/s41598-017-04262-y

**Published:** 2017-06-20

**Authors:** Thiago Rodrigues Dutra, Valéria Monteze Guimarães, Ednilson Mascarenhas Varela, Lílian da Silva Fialho, Adriane Maria Ferreira Milagres, Daniel Luciano Falkoski, José Cola Zanuncio, Sebastião Tavares de Rezende

**Affiliations:** 1 Departamento de Bioquímica e Biologia Molecular, Universidade Federal de Viçosa, 36570-000 Viçosa, Minas Gerais Brazil; 2 Departamento de Biotecnologia, Escola de Engenharia de Lorena, Universidade de São Paulo, 12602-810 Lorena, SP Brazil; 3 Novozymes Latin America - R. Prof. Francisco Ribeiro, 683 - Barigui, Araucária, PR - 83707-660 Brazil; 4 Departamento de Entomologia/BIOAGRO, Universidade Federal de Viçosa, Minas Gerais, 36570-900 Brazil

## Abstract

Low cost and high efficiency cellulolytic cocktails can consolidate lignocellulosic ethanol technologies. Sugarcane bagasse (SCB) is a low cost agro-industrial residue, and its use as a carbon source can reduce the costs of fungi cultivation for enzyme production. *Chrysoporthe cubensis* grown under solid state fermentation (SSF) with wheat bran has potential to produce efficient enzymatic extracts for SCB saccharification. This fungus was grown under submersed fermentation (SmF) and SSF with *in natura* SCB, pretreated with acid or alkali and with others carbon sources. *In natura* SCB induced the highest carboxymethylcellulase (CMCase), xylanase, β-xylosidase, α-galactosidase and mannanase activities by *C*. *cubensis* under SSF. *In natura* and washed SCB, inducers of enzyme production under SSF, did not induce high cellulases and hemicellulases production by *C*. *cubensis* in SmF. The *C*. *cubensis* enzymatic extract produced under SSF with *in natura* SCB as a carbon source was more efficient for lignocelulolic biomass hydrolysis than extracts produced under SSF with wheat bran and commercial cellulolytic extract. *Chrysoporthe cubensis* showed high potential for cellulases and hemicellulases production, especially when grown under SSF with *in natura* SCB as carbon source.

## Introduction

Plant biomass conversion to biofuels is a key strategy to replace fossil fuels by cleaner sources as part of the global energy chain^1^. Nature produces around 200 billion tons of lignocellulosic biomass per year with energy content about 10 times the annual world consumption^2^.

Plant biomass degradation to monomeric sugars produces raw materials which can be converted to products such as ethanol, lactic acid, sorbitol, xylitol and others^2, 3^. Efficient hydrolysis of lignocellulosic material requires complete enzyme cocktails, rich in cellulases, hemicellulases, ligninases and auxiliary enzymes^4, 5^.

Fungi can efficiently degrade biomass because this carbon source predominates in their natural biotopes^6^. Full cellulose depolymerization requires synergistic action of the cellulases endoglucanase (EC 3.2.1.4), cellobiohydrolase (EC 3.2.1.176) and β-glucosidase (EC 3.2.1.21). The hemicellulose fraction, a more complex polymer, requires a more diverse group of enzymes, the hemicellulases. Complete enzymatic hydrolysis of xylan, the main hemicellulose polymer, requires β-1,4-endo-xylanase (EC 3.2.1.8), β-xylosidase (EC 3.2.1.37), and ancillary enzymes, as α-arabinofuranosidase (EC 3.2. 1:55), α-glucuronidase (EC 3.2.1.139), α-galactosidase (EC 3.2.1.22), acetyl xylan esterase (EC 3.1.1.72) and ferulic acid esterase (EC 3.1.1.73)^7, 8^.

The high cost of cellulolytic enzymes is a major bottleneck to lignocellulosic ethanol production. The use of agricultural residues, such as sugarcane bagasse, is an alternative to reduce enzyme production costs during microorganism cultivation. Brazil is the largest sugarcane producer in the world, producing over 200 million tons of sugarcane bagasse per year^9^. The use of this raw material in biotechnological processes is interesting due of its low cost and high abundance^10^.

Commercial companies use a small number of ascomycetes fungi (*Trichoderma reesei* and several species of *Aspergillus*) to produce cellulolytic enzyme cocktails^6^. However, other fungi genera such as *Fusarium*, *Penicillium* and *Chrysoporthe* have been studied for this purpose^3, 5, 11, 12^.

The ascomycete fungus, a *Eucalyptus* pathogen, *Chrysoporthe cubensis* cultured on solid medium with wheat bran produced a more efficient enzymatic extract for sugarcane bagasse saccharification than commercial cellulolytic preparations^2, 13^. Extracts produced by this fungus exhibited specific activities of endoglucanase, β-glucosidase, β-xylosidase and pectinase higher than those of a commercial cellulolytic extract^13^.

The objective of this study was to obtain cellulase and hemicellulase production by *C*. *cubensis* grown under solid (SSF) and submerged (SmF) state fermentation. The *Chrysoporthe cubensis* extract produced under SSF with SCB was applied to saccharification processes and its performance was compared with that of the extract produced by this fungus under SSF with wheat bran and a commercial extract.

## Results and Discussion

### Chemical composition of the materials

Cellulose, hemicellulose and lignin concentrations in the *in natura* sugarcane bagasse (SCB) were 42.4%, 28.8% and 24.0%, respectively. Additionally, sucrose and xylose concentrations were 10.3 and 1.94 mg g^−1^ (w/w), respectively. Washing with distilled water removed soluble sugars, sucrose and xylose from this material (Table 1).

**Table 1:** Chemical composition of sugarcane bagasse (SCB) samples in comparison to chemical of wheat bran^46^.

Biomass	Lig	Cel (%)	Hemi	Protein	Glu	Suc	Xylo
				(mg g^−1^ dry biomass)			
*In natura* SCB	24.0 ± 0.3^b^	42.4 ± 0.2^c^	28.8 ± 0.7^a^	—	ND	10, 3	1, 9
Washed SCB	24.0 ± 0.3^b^	42.4 ± 0.2^c^	28.8 ± 0.7ª	—	ND	ND	ND
Alkali pretreated SCB	11.7 ± 0.7^c^	53.6 ± 0.3ª	28.8 ± 0.5ª	—	ND	ND	ND
Acid pretreated SCB	33.6 ± 0.2ª	51.1 ± 0.6^b^	8.2 ± 0.3^b^	—	ND	ND	ND
Wheat bran	8.2	43.7	28.2	2.4	—	—	—

Lig: Lignin; Cel: cellulose; Hemi: hemicellulose; Glu: glucose; Suc: sucrose; Xylo: xylose. Averages followed by the same letter do not differ by the Tukey test at 5% significance.

Chemical composition of alkali pretreated sugarcane bagasse SCB consisted of 53.6%, 28.8% and 8.0% of cellulose, hemicellulose and lignin, respectively. On the other hand, acid pretreated SCB showed 51.1, 8.2 and 33.6%, respectively. These results agreed with those reporting that pretreatments with dilute acid remove hemicellulose and alkaline pretreatments remove lignin from lignocellulosic biomass^14^.

### Enzymatic production by *Chrysoporthe cubensis* under submerged fermentation (SmF)

Cellulase and hemicellulase enzymes production by *C*. *cubensis* under submerged fermentation (SmF) allowed to evaluate the effect of cell wall components (e.g., xylan, pectin) and simpler sugars (e.g. glucose, cellobiose) on regulating production of cellulolytic enzymes by this fungus.

Wheat bran, acid pretreated SCB and carboxymethylcellulose (CMC) induced the highest CMCase productions by *C*. *cubensis*, with 3.4, 2.7 and 2.6 U mL^−1^, respectively, whilst CMC, wheat bran and pectin induced the highest β-glucosidase productions by this fungus, with 0.5, 0.4 and 0.3 U mL^−1^, respectively (Table 2). Alkali pretreated SCB, wheat bran, acid pretreated SCB, CMC and xylan induced production of the highest xylanase activities under SmF, 37.2, 14.2, 11.0, 10.2 and 10.1 U mL^−1^, respectively. The fact of CMC induced CMCase, β-glucosidase and xylanase enzyme activities of 2.6, 0.47 and 10.2 U mL^−1^ by *C*. *cubensis*, respectively, differed from results for *Aspergillus* genus fungi that produce xylan-degrading enzymes in the presence of substrates containing xylose and xylan, but not with cellulose^15^. CMCase and xylanase production by *C*. *cubensis* cultured in the medium with CMC indicates that, as occurs in *T*. *reesei*, this carbon source is involved in the activation of cellulase and hemicellulase production. *Trichoderma reesei* has the transcription factor ACEII that actives cellulase and hemicellulase production pathways in the presence of celullose^16^. This suggests that *C*. *cubensis* may have transcription factors with similar role to the ACEII of *T*. *reesei*.

**Table 2:** Cellulase and hemicelulase activities produced by *Chrysoporthe cubensis* under submerged fermentation with different carbon sources (Carb.).

Carb.	Activity (U mL^−1^)						
	CMCase	β-Glu	Xln	β-Xil	α-Gal	α-Ara	MN
IN-SCB	0.12 ± 0.020^d^	0.02 ± 0.001^de^	0.35 ± 0.030^c^	ND	0.03 ± 0.001^b^	0.02 ± 0.002^b^	0.13 ± 0.013^def^
W-SCB	0.33 ± 0.040^d^	ND	1.56 ± 0.090^c^	ND	0.01 ± 0.001^c^	ND	0.10 ± 0.002^ef^
Alk-SCB	2.10 ± 0.550^c^	0.03 ± 0.009^de^	37.20 ± 7.41ª	0.10 ± 0.023ª	ND	0.01 ± 0.001^b^	0.22 ± 0.044^bc^
Ac-SCB	2.70 ± 0.050^b^	0.13 ± 0.004^c^	11.00 ± 0.750^b^	0.04 ± 0.001^b^	0.02 ± 0.002^c^	ND	0.41 ± 0.020ª
WBran	3.36 ± 0.010ª	0.36 ± 0.027^b^	14.20 ± 0.690^b^	0.02 ± 0.002^bc^	ND	0.05 ± 0.004ª	0.15 ± 0.020^cde^
LBGum	0.04 ± 0.006^d^	0.02 ± 0.001^de^	0.30 ± 0.030^c^	ND	0.01 ± 0.000^*c^	ND	0.07 ± 0.003^f^
CMC	2.60 ± 0.074^bc^	0.47 ± 0.018ª	10.20 ± 0.581^b^	0.01 ± 0.000^*c^	0.01 ± 0.001^c^	0.01 ± 0.000^*b^	0.12 ± 0.000^*def^
Pectin	0.05 ± 0.005^d^	0.30 ± 0.023^b^	0.56 ± 0.072^c^	0.01 ± 0.001^c^	0.06 ± 0.011^a^	ND	0.13 ± 0.013^def^
Xylan	0.30 ± 0.042^d^	0.14 ± 0.063^c^	10.10 ± 0.850^b^	ND	ND	ND	ND
Lactose	0.30 ± 0.018^d^	0.03 ± 0.006^de^	ND	ND	ND	ND	0.19 ± 0.050^cd^
Cellobiose	0.02 ± 0.004^d^	ND	0.22 ± 0.054^c^	ND	ND	ND	ND
Galactose	0.22 ± 0.012^d^	ND	0.53 ± 0.022^c^	ND	ND	ND	ND
Arabinose	0.22 ± 0.017^d^	0.01 ± 0.000^*de^	1.35 ± 0.022^c^	ND	ND	ND	0.12 ± 0.040^def^
Xylose	0.33 ± 0.003^d^	0.10 ± 0.006^d^	1.65 ± 0.164^c^	ND	ND	ND	0.31 ± 0.030^b^
Mannose	0.02 ± 0.005^d^	ND	0.14^c^	ND	ND	ND	ND
Glucose	0.02 ± 0.005^d^	ND	0.10^c^	ND	ND	ND	ND

SCB = sugarcane bagasse; IN-SCB = *in natura* sugarcane bagasse; W-SCB = washed SCB; Alk-SCB = alkali pretreated SCB; Ac-SCB = acid pretreated SCB; WBran = wheat bran, LBGum = locust bean gum. CMCase = carboxymethylcellulase; β-Glu = β-glicosidase; Xyl = xylanase; β-Xy l = β-xylosidase; α-Ara = α-arabinofuranosidase; α-Gal = α-galactosidase; MN = Mannanase; ND = not detected. Averages followed by the same letter do not differ by the Tukey test at 5% of significance. *Standard deviations lower than 0.001.

Xylan induced higher xylanase production (10.1 U mL^−1^) by *C*. *cubensis* compared to CMCase production (0.3 U mL^−1^), agreeing with results for *Fusarium graminearum*. This fungus regulates xylanase production with transcriptional activator *Xyr1* involved in activation of gene transcription to xylanases, but not cellulase^17^. *Xyr1* orthologous genes are highly conserved in ascomycetes, suggesting that similar signaling pathways may occur in *C*. *cubensis*, an ascomycete^18, 19^.

Alkali pretreated SCB induced production of the highest xylanase activity (37.2 U mL^−1^) by *C*. *cubensis* under SmF among the carbon sources evaluated. This can be explained by the fact that the alkaline pretreatment mainly solubilizes lignin from biomass, promoting a relative increase in the concentration of cellulose and hemicellulose^20^, inducers of xylanase production by this fungus (Table 2).

Glucose, xylose, lactose, galactose, arabinose and mannose did not induce cellulase and hemicellulase production by *C*. *cubensis*. These results are in agreement with reports that in the presence of preferred carbon sources the cellulase and hemicellulase production in fungi was inhibited by carbon catabolite repression^15^.

### Enzyme production by *Chrysoporthe cubensis* under solid-state fermentation (SSF)

CMCase, xylanase, β-xylosidase, α-galactosidase, and mannanase activities of enzymatic cocktail of *C*. *cubensis* grown under solid state fermentation (SSF) with *in natura* SCB as the carbon source showed activities of 33.2, 602, 2.0, 2.4 and 7.1 U g^−1^, respectively. These activities were the largest ones produced by this fungus on all tested carbon sources. The β-glucosidase and α-arabinofuranosidase activities for cultivation on this substrate were 1.5 and 8.2 U/g respectively (Table 3). *Chrysoporthe cubensis* cultured on washed SCB produced lower CMCase, xylanase, β-xylosidase, α-galactosidase, α-arabinofuranosidase and mannanase activities (17.9, 500, 0.6, 0.4, 1.8 and 3.2 U g^−1^, respectively) than those obtained by the cultivation on *in natura* SCB (Table 2). This differs from results for *Trichoderma reesei*, which showed higher enzyme production in cultivation with washed SCB compared to non-washed SCB^21^. *Trichoderma reesei* grown on *in natura* SCB with high glucose content showed lower enzyme production compared to that cultivated on washed, glucose-free SCB^21^. The greater induction of cellulase and hemicellulase production by *C*. *cubensis* when cultivated on *in natura* SCB compared to washed SCB can be explained by the absence of detectable glucose levels in the former SCB (Table 1). Glucose is a repressor molecule of cellulase and hemicellulase production by fungi, and its absence in the *in natura* SCB used for *C*. *cubensis* cultivation contributed to higher production in the washed material^22, 23^. Moreover, the presence of xylose in the *in natura* SCB presented an activating effect on enzyme production compared to cultivation with washed SCB. This confirms the role of this molecule as an activator of cellulase and hemicellulase production in fungi^24^. *In natura* SCB contained sucrose (30.1 mg g^−1^ of dry SCB mass), a repressor of cellulase production in filamentous fungi, even in the presence of lignocellulosic material^25^. However, the sucrose concentrations found in the *in natura* SCB did not suppress cellulolytic production by *C*. *cubensis*. The xylanase, α-arabinofuranosidase, β-glucosidase and CMCase activities produced by *C*. *cubensis* under SSF with *in natura* SCB were higher than those obtained by fungi of the *Trichoderma* genus used to produce commercial cellulolytic preparations, enhancing the potential for hydrolytic enzymes production by *C*. *cubensis* on *in natura* SCB^26^. *In natura* SCB was a better cellulase and hemicellulase production inducer by *C*. *cubensis* than wheat bran, the standard carbon source for enzyme production by this fungus^3, 5, 13^. This is an interesting result considering that *in natura* SCB is an abundant and inexpensive residue, does not require pretreatment or washing, and can be used in *on site* enzyme production in sugar and alcohol industries^9, 27^.

**Table 3:** Activities produced by *Chrysoporthe cubensis* under solid state fermentation (SSF) with different carbon sources (Carb.).

Carb.	Activity (U g^−1^ dry substrate)						
	CMCase	β-Glu	Xln	β-Xyl	α-Gal	α-Ara	MN
*In*-SCB	33.2 ± 0.42ª	1.5 ± 0.04^bc^	602 ± 17.6^a^	2.0 ± 0.02^a^	2.4 ± 0.02^a^	8.2 ± 0.04^b^	7.1 ± 0.23^a^
W-SCB	17.9 ± 0.72^b^	1.4 ± 0.06^c^	500 ± 9.1^b^	0.6 ± 0.09^b^	0.4 ± 0.03^b^	1.8 ± 0.12^c^	3.2 ± 0.60^b^
Alk-SCB	0.6 ± 0.03^e^	0.3 ± 0.01^d^	26 ± 6.9^e^	ND	0.2 ± 0.02^c^	1.5 ± 0.15 ^cd^	0.6 ± 0.04^e^
Ac-SCB	2.5 ± 0.04^d^	ND	220 ± 0.8^d^	ND	ND	ND	ND
WBran	16.5 ± 0.83^b^	2.1 ± 0.05^a^	322 ± 0.8^c^	0.1 ± 0.02^c^	0.2 ± 0.02^c^	0.9 ± 0.01^c^	1.5 ± 0.07^d^
LBGum	9.3 ± 0.37^c^	1.6 ± 0.13^b^	20 ± 2.2^e^	0.6 ± 0.01^b^	0.3 ± 0.02^c^	16.5 ± 1.10ª	2.4 ± 0.12^c^

SCB = sugarcane bagasse; *In*-SCB = *in natura* SCB; W-SCB = washed SCB; Alk-SCB = alkali pretreated SCB, Ac-SCB = acid pretreated SCB; WBran = wheat bran; LBGum = locust bean gum. CMCase = carboxymethylcellulase; β-Glu = β-glicosidase; Xyl = xylanase; β-Xyl = β-xylosidase; α-Ara = α-arabinofuranosidase; α-Gal = α-galactosidase; MN = Mannanase; ND = not detected. Averages followed by the same letter do not differ by the Tukey test at 5% of significance.

Acid pretreated SCB induced xylanase production (220 U g^−1^), but this material was a weak inducer of CMCase activity (2.5 U g^−1^), and did not induce β-glucosidase production by this *C*. *cubensis*, although this carbon source presents a high cellulose content. This finding is in disagreement with the report that cellulose is a strong cellulase production inducer in filamentous fungi^28^. Acid pretreated SCB had higher cellulose content than non-pretreated SCB, and therefore it was expected that this biomass would induce cellulase production by *C*. *cubensis*. This result further demonstrates the potential of this enzyme cocktail for pulp biobleaching processes. In these processes cellulase-poor xylanase cocktails act in the depolymerization of hemicelluloses precipitated on the pulp fiber surface and remove carbohydrate-lignin complexes generated during the Kraft process, without harming the cellulose fiber^29–31^.

Locust bean gum (galactomannan) activated α-arabinofuranosidase production by *C*. *cubensis* under SSF, inducing higher values of this activity (16.5 U g^−1^) than the other carbon sources. The induction of α-arabinofuranosidase production by galactomannans in filamentous fungi under SSF is not well known. High enzyme production by *C*. *cubensis* under SSF with locust bean gum is in agreement with results obtained with *T*. *reesei* which reported CMCase, xylanase and mannanase production activated with this carbon source^32^. α-Arabinofuranosidases assist hemicellulose hydrolysis in the biomass saccharification process, since they catalyze the hydrolysis of α-arabinose bonds linking residues in the backbone of hemicellulose. These enzymes can be used to improve wine flavor, the main feature of this product^33, 34^.

### Specific activities of the extracts produced by *C*. *cubensis* under SSF and SmF with SCB

The specific activities of cellulases and hemicellulases from *C*. *cubensis* extracts cultured with SCB under SSF and SmF demonstrated that this fungus is a better cellulolytic enzyme producer under SSF than under SmF with *in nature* and washed SCB as carbon sources (Table 4

**Table 4:** Specific activities of cellulases and hemicellulases produced by *Chrysoporthe cubensis* under solid state fermentation (SSF) and submerged fermentation (SmF) with sugarcane bagasse (SCB).

		Specific activity (U mg^−1^)						
		CMCase	β-Glu	Xln	β-Xyl	α-Gal	α-Ara	MN
SSF	*In*-SCB	27.6 ± 0.5ª	1.3 ± 0.06^b^	502 ± 30^ab^	1.7 ± 0.08ª	2.0 ± 0.06^a^	6.8 ± 0.9ª	5.9 ± 0.4ª
	W-SCB	20.2 ± 0.6^b^	1.5 ± 0.08ª	551 ± 22^a^	0.7 ± 0.07^b^	0.5 ± 0.12^b^	2.0 ± 0.1^b^	3.5 ± 0.2^b^
	Alk-SCB	2.2 ± 0.1^f^	1.1 ± 0.10^c^	221 ± 4^c^	ND	0.3 ± 0.09^b^	4.9 ± 0.6ª	2.3 ± 0.3^cd^
	Ac-SCB	5.7 ± 0.3^e^	ND	489 ± 2^b^	ND	ND	ND	ND
SmF	*In*-SCB	1.9 ± 0.3^f^	0.03 ± 0.00^*,d^	5.6 ± 0.4^e^	ND	0.6 ± 0.01^b^	ND	2.1 ± 0.2^cd^
	W-SCB	5.1 ± 0.7^e^	0.02 ± 0.00^*,d^	26.5 ± 1.5^de^	ND	0.1 ± 0.00^*c^	ND	1.7 ± 0.0^*de^
	Alk-SCB	11.5 ± 1.3^d^	0.03 ± 0.01^d^	185 ± 13^c^	ND	ND	ND	1.1 ± 0.2^e^
	Ac-SCB	16.9 ± 0.3^c^	0.13 ± 0.01^d^	68.9 ± 5^d^	ND	0.1 ± 0.03^c^	ND	2.6 ± 0.1^c^

SCB = sugarcane bagasse; *In*-SCB = *in natura* SCB; W-SCB = washed SCB; Alk-SCB = alkali pretreated SCB, Ac-SCB = acid pretreated SCB. CMCase = carboxymethylcellulase; β-Glu = β-glicosidase; Xyl = xylanase; β-Xyl = β-xylosidase; α-Ara = α-arabinofuranosidase; α-Gal = α-galactosidase; MN = Mannanase; ND = not detected. The averages followed by the same letter do not differ significantly by the Tukey test at 5% of significance. *Standard deviations lower than 0.01.

).

The production of enzyme cocktails with specific CMCase, β-glucosidase and xylanase activities generated by *C*. *cubensis* grown in SSF conditions with *in natura* SCB were 14.5, 4.3 and 90 times higher than those of the cocktails produced under SmF with the same material, respectively. The better cellulolytic production by *C*. *cubensis* on *in natura* SCB under SSF compared to SmF indicated advantages of its cultivation, because this process requires less water, no agitation and less energy demand^35^.

*Chrysoporthe cubensis* extract produced with alkali and acid pretreated SCB under SSF had higher specific xylanase activities (221 and 489 U mg^−1^, respectively) than those of the extracts produced under SmF with the same carbon sources (185 and 68.9 U/mg, respectively) (Table 4). Moreover, the extracts produced by this fungus with alkali and acid pretreated SCB under SmF showed higher specific activities of CMCase (11.5 and 16.9 U mg^−1^, respectively) than those produced under SSF (2.2 and 5.7 U mg^−1^ respectively). These results indicate that the best culture condition (SSF or SmF) for both cellulase and hemicellulase production by *C*. *cubensis* varies with the carbon source as reported for *Lentinusedodes* and *Pleurotus* species^36^.

### *Chrysoporthe cubensis* enzymatic profile and biomass enzymatic saccharification

The *Chrysoporthe cubensis* extract produced under SSF with *in natura* SCB was applied to saccharification processes and compared to that produced by this fungus under SSF with wheat bran, the standard substrate used for enzyme production by this fungus^3, 13^, as well as a commercial extract. To compare the extracts applied in saccharification assays, the enzymatic activities were normalized relative to FPase activity, which is the total cellulose activity of the enzymatic complexes (Table 5). The β-glycosidase/FPase ratios of the *C*. *cubensis* extracts were similar to those of the commercial extract Multifect CL^3^, but the Multifect CL extract had a CMCase/FPase ratio 3.1 and 4.3 times higher than that of the extracts produced by *C*. *cubensis* under SSF with in nature SCB and wheat bran, respectively (Table 5).

**Table 5:** Comparative analysis of cellulase and hemicellulase activities of the extracts produced by *Chrysoporthe cubensis* under SSF with *in natura* sugarcane bagasse (SCB) or wheat bran in comparison to Multifect CL^3^.

Enzyme	Units of enzymatic activity/Units of FPase activity		
	*In natura* SCB	Wheat bran	Multifect CL
FPase	1.0 ± 0.02	1.0 ± 0.05	1.0
CMCase	17.7 ± 0.3	12.6 ± 0.5	54.1
β-Glu	0.8 ± 0.02	1.6 ± 0.03	0.8
Xylanase	321.0 ± 25	245.9 ± 4.7	32.8
β-Xylosidase	1.1 ± 0.08	0.4 ± 0.01	ND
α-Ara	4.3 ± 0.09	0.7 ± 0.01	ND
α-Gal	1.3 ± 0.02	0.2 ± 0.01	ND
Mannanase	3.8 ± 0.3	1.1 ± 0.05	2.0

The values displayed were obtained dividing each total enzymatic activity by total FPase activity found in each cellulolytic extract.

The *Chrysoporthe cubensis* extracts showed greater complexity of hemicelulolitic activities than the commercial product Multifect CL. This fungus secreted β-xilosidade, α-arabinofuranosidase and α-galactosidase, enzymes absent in the commercial extract. Furthermore, *C*. *cubensis* extracts showed xylanase/FPase ratios 9.8 and 7.5 times higher when grown with *in natura* SCB and wheat bran under SSF than the commercial enzyme extract, respectively. This result is interesting because the hemicellulases, xylanases, α-arabinofuranosidase and β-xylosidase are important in the saccharification of lignocellulosic materials such as SCB, which are rich in hemicelluloses^32, 37^. The absence or lack of these enzymes limits the cellulases action in cellulose fiber hydrolysis, because the hemicellulases acts synergistically with cellulases in the hydrolysis of lignocellulosic materials^38^.

**Figure 1:**
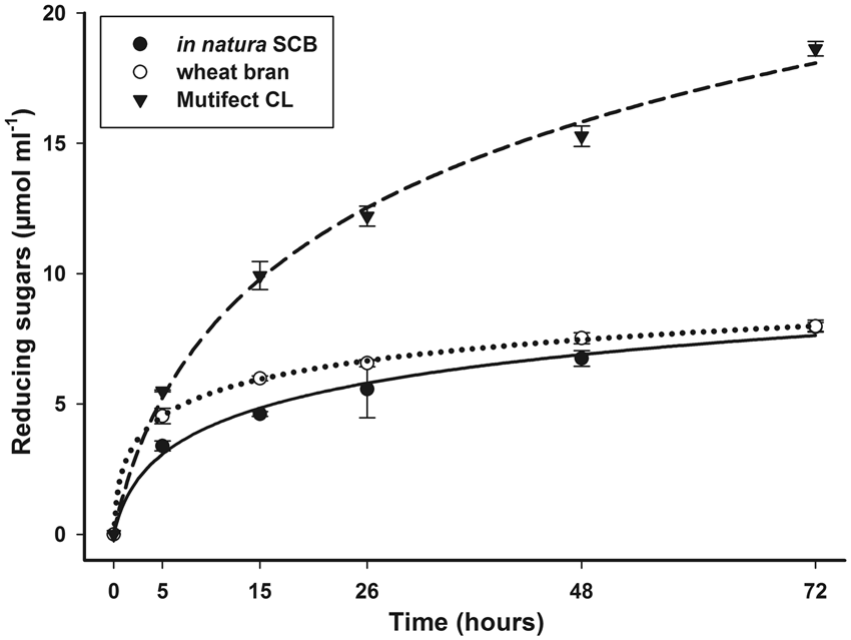
Production of reducing sugars per time by enzymatic saccharification of microcrystalline cellulose Avicel using extracts produced by *Chrysoporthe cubensis* under SSF with *in natura* sugarcane bagasse (SCB) (●) or wheat bran (○) and commercial extract Multifect CL (▼).

The commercial extract Multifect more efficiently released reducing sugars (18.6 μmol ml^−1^) than the extracts produced by *C*. *cubensis* under SSF with wheat bran and *in natura* SCB (7.95 and 7.98 μmol/mL, respectively) in saccharification assays of the microcrystalline cellulose Avicel (Fig. 1). This can be justified by the higher CMCase/FPase ratio presented by the commercial cocktail compared to *C*. *cubensis* extracts, since endoglucanases start the saccharification process hydrolyzing the inner parts of the cellulose fiber, releasing smaller chains which are hydrolyzed by cellobiohydrolases^39^.

**Figure 2:**
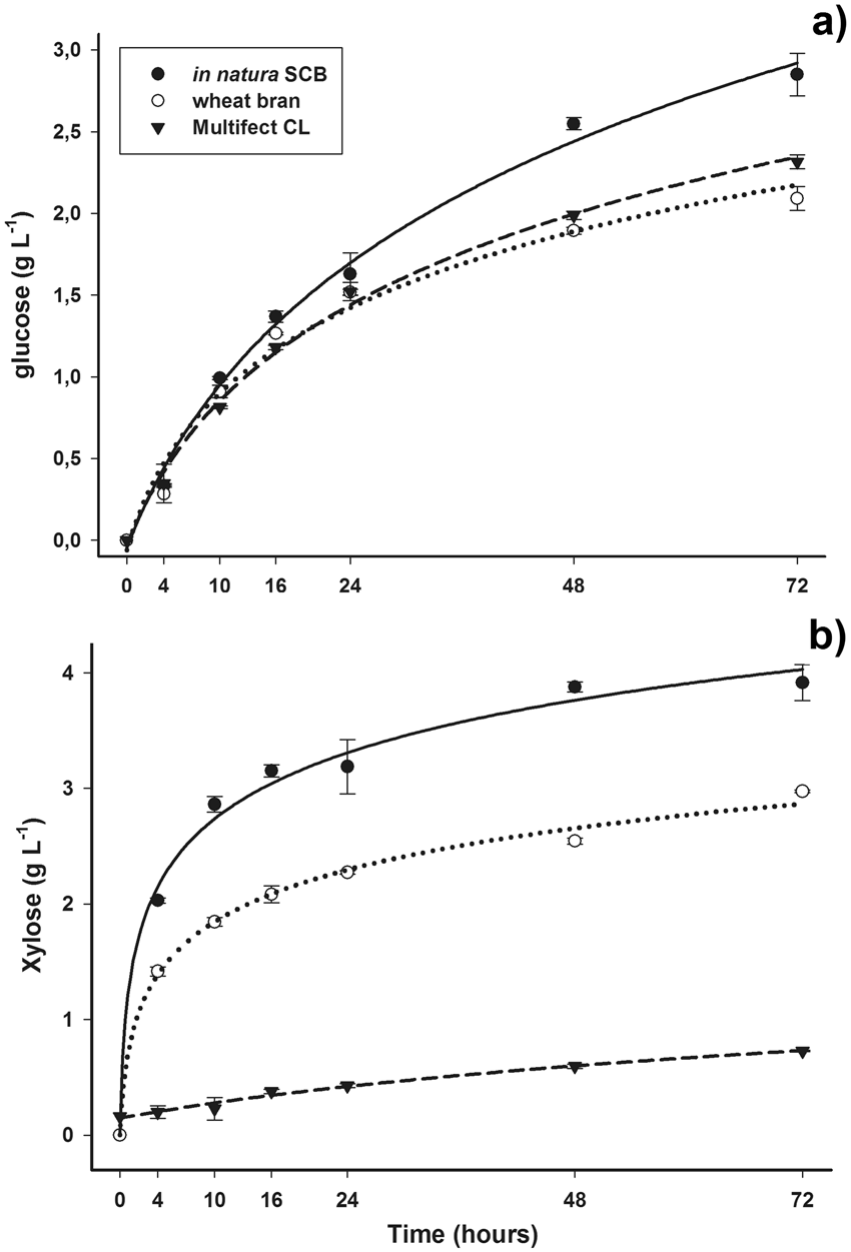
Glucose (**a**) and xylose (**b**) production per time by enzymatic saccharification of alkali pretreated sugarcane bagasse (SCB) using extracts produced by *Chrysoporthe cubensis* under solid state fermentation (SSF) with *in natura* SCB (●) or wheat bran (○) and commercial extract Multifect CL (▼).

The cocktail produced by *C*. *cubensis* under SSF with *in natura* SCB released more glucose (2.8 g L^−1^) in saccharification of alkali pretreated SCB than the commercial extract and that the extract produced by this fungus under SSF with wheat bran, 2.3 and 1.98 g L^−1^, respectively (Fig. 2a). The extract produced by *C*. *cubensis* under SSF with *in natura* SCB released more xylose from the alkali pretreated SCB (3.91 g L^−1^ xylose) than the commercial extract and the extract from this fungus cultivated under SSF with wheat bran (0.72 and 2.97 g L^−1^ xylose, respectively) after 72 hours of saccharification (Fig. 2b).

Greater effectiveness of the *C*. *cubensis* extract produced under SSF with *in natura* SCB than that of the commercial extract regarding glucan and xylan hydrolysis was mainly due to greater richness of hemicellulase activities in the *C*. *cubensis* extract compared to the commercial cocktail. The hydrolysis of hemicellulose by xylanase, α-arabinofuranosidase, β-xylosidase, mannanase and α-galactosidase enzymes from *C*. *cubensis* facilitates the access of cellulase enzymes to cellulose. Hemicellulose acts as a barrier, preventing access of cellulases to cellulose, and the hydrolysis of this polymer is essential for efficient saccharification of plant biomass^40^.

The superior ability for saccharification of alkali pretreated SCB by the *C*. *cubensis* extract produced under SSF with *in natura* SCB in relation to that produced on wheat bran agrees with the results reported for *Aspergillus niger* and *T*. *reesei*, which produced more efficient enzyme extracts for SCB saccharification when grown with this substrate than with wheat bran^19^. This is due to the fact that fungi can produce enzyme complexes adjusted to the presents fractions in the biomass used to cultivate this fungus^25^.

*Chrysoporthe cubensis* cultured under SSF with *in natura* SCB as the sole carbon source produced a more effective enzymatic extract for saccharification of alkali pretreated SCB than that produced by the same fungus cultivated on a standard carbon source (wheat bran) as well as the commercial cellulolytic extract. This extract showed better saccharification performance of alkali pretreated SCB due to a more complete hemicellulolytic arsenal, demonstrating the importance of these enzymes in saccharification of lignocellulosic materials.

## Conclusions

Enzyme production by *Chrysoporthe cubensis* cultured with sugarcane bagasse (SCB) was studied for the first time, and this carbon source can be used to efficiently produce cellulases and hemicellulases by this fungus. The enzyme production by *C*. *cubensis* with *in natura* SCB is important because this is a low-cost material, does not require washing, can be used for enzyme production by the sugar and alcohol industry and it induced production of an enzyme cocktail production which was effective for SCB saccharification.

## Methods

### Biomass composition analysis

The chemical composition of the *in natura*, washed, acid pretreated and alkali pretreated SCB were determined using a modified Klason lignin method derived from the TAPPI Standard Method T222 om-98^41^. Extractive-free biomass (0.3 g) was incubated at 30 °C with 3 mL of 72% H2SO4 for 1 h with occasional mixing. The slurry was then transferred into a penicillin bottle containing 84 mL of deionized water and the flask sealed with a rubber stopper and aluminum seal. The bottle was placed in an autoclave calibrated at 118 °C for 1 h, then the slurry was filtered through a medium coarseness sintered glass filter for gravimetric determination of acid-insoluble lignin. Concentrations of biomass sugars (arabinose, galactose, glucose, xylose, and mannose) in the filtrate were quantified using high-performance liquid chromatography (HPLC), while acid-soluble lignin was determined by absorption measurements at 205 nm^42^. The HPLC system Dionex DX-300 (Dionex Co. – Sunnyvale, CA, USA) was equipped with a Carbopac PA1 column and a pulsed amperometric detector with a gold electrode. Prior to injection, samples were filtered through 0.45-mm HV filters and a volume of 20 μL loaded into the chromatograph system. The column was pre-equilibrated with a NaOH solution, 300 mM, and elution was carried out at a flow rate of 1.0 mL min^−1^ at room temperature.

### Biomass washing and pretreatment

Sugarcane bagasse (SCB) was provided by the Jatiboca Sugar and Alcohol Plant in Urucânia, Minas Gerais state, Brazil. This material was dried, ground (particle size less than 1 mm) and stored at −80 °C for further use. Four types of SCB were obtained from the sample described: washed SCB, acid pretreated SCB, alkali pretreated SCB and *in natura* SCB, which was not subjected to washing or pretreatments. The washed SCB was obtained by washing of the *in natura* SCB with distilled water until the contents of glucose, xylose and sucrose were not detectable by HPLC analysis. This material was dried at 70 °C until reaching constant weight. NaOH 1% (w/v) or H_2_SO_4_ 1% (w/v) were used to pretreat 25 g of *in natura* SCB with 10% (w/v) solids loading, generating the alkali pretreated SCB and the acid pretreated SCB, respectively. The pretreatments were performed in an autoclave at 120 °C for 60 min. These pretreated materials were separated into solid and liquid fractions using a Buchner funnel fitted with filter paper. The solid fraction was washed thoroughly with distilled water, sealed in a hermetic vessel to retain moisture and stored at −20 °C.

### Microorganism

The fungus *C*. *cubensis* LPF-1 used in this study was obtained from the mycological collection of the Forest Pathology Laboratory of the Universidade Federal de Viçosa in Viçosa, Minas Gerais State, Brazil. The fungus was maintained on PDA (potato dextrose agar) plates at 28 °C and subcultured periodically.

### Submerged fermentation (SmF)

*Chrysoporthe cubensis* was grown in liquid medium using 1% (w/v) glucose, mannose, xylose, arabinose, galactose, cellobiose, lactose, xylanbirch wood, pectin, locust bean gum, carboxymethylcellulose, wheat bran, acid pretreated SCB, alkali pretreated SCB, washed SCB and *in natura* SCB as carbon sources. Cultivation was conducted in 125 mL Erlenmeyer flasks with 50 mL of culture medium composed of (g L^−1^): (NH_4_)_2_SO_4_, 1.4; urea, 0.3 g; KH_2_PO_4_, 2.0; MgSO_4_ 7H2O, 0.3; CaCl_2_, 0.3; and yeast extract, 2.0. The carbon source was added tothe medium at concentration of 1% (w/v). Trace elements FeSO_4_ 7H_2_O (1.0 mg L^−1^), ZnCl_2_ (3.5 mg L^−1^), MnSO_4_ H_2_O (1.0 mg L^−1^), CoCl 6H_2_O (1.0 mg L^−1^), CuSO_4_. 5H_2_O (0.5 mg L^−1^) and 20MoO_3_ 2H_3_PO_4_ 48H_2_O (0.02 mg L^−1^) were also added.

The flasks were autoclaved at 120 °C for 20 minutes, inoculated with 0.5 mL of a spore suspension (2.2 × 10^6^ spores mL^−1^) and placed in a shaker for seven days at 180 rpm and 28 °C. The samples were centrifuged at 10,000 × g for 20 minutes and the supernatant used as enzyme extracts.

### Inoculum preparation for solid state fermentation (SSF)

The inoculum was prepared by growing the fungus under submerged fermentation (SmF) in 250 mL Erlenmeyer flasks containing 100 mL of medium with the following composition, in g L^−1^: glucose, 10.0; NH_4_NO_3_, 1.0; KH_2_PO_4_, 1.0; MgSO_4_, 0.5 and yeast extract, 2.0. Each flask was inoculated with 1.0 ml agar plugs cut from a 5 day-old colony of *C*. *cubensis* grown on PDA plates and incubated in a rotary shaker for 5 days, at 150 rpm and 28 °C. The culture obtained was used to inoculate the solid culture media.

### Solid state fermentation (SSF)

*Chrysoporthe cubensis* was grown under SSF to evaluate the effect of different carbon sources on enzyme production in this cultivation condition. *Chrysoporthe cubensis* was cultured under solid state fermentation (SSF) using washed and *in natura* sugarcane bagasse (SCB), acid and alkali pretreated SCB, wheat bran and locust bean gum as the main carbon source. The fermentations were carried out in 125 mL Erlenmeyer flasks containing 5 g (dry weight) of the substrate moistened with culture media presenting the following composition, in g L^−1^: NH_4_NO_3_, 1.0; KH_2_PO_4_, 1.5; MgSO_4_, 0.5; CuSO_4_, 0.25 and yeast extract, 2. Furthermore, MnCl_2_ (0.1 mg L^−1^), H_3_BO_3_ (0.075 mg L^−1^), Na_2_MoO_4_ (0.02 mg L^−1^), FeCl_3_ (1.0 mg L^−1^) and ZnSO_4_ (3.5 mg L^−1^) also were added to the medium as trace elements.

The assays consisted of cultivation with washed SCB, *in natura* SCB (non-washed SCB), acid pretreated SCB, alkali pretreated SCB, wheat bran and locust bean gum with final moisture contents of 80, 80, 90, 90, 75 and 60%, respectively. The flasks were autoclaved at 120 °C for 20 min and then inoculated with 3 mL (containing 1.5 × 10^7^ spores mL^−1^) of the inoculum obtained as aforementioned. The flasks were maintained at 30 °C in a temperature controlled chamber and the enzymatic extraction performed after seven days of fermentation. Enzymes secreted during SSF were extracted with sodium acetate buffer, 50 mM, pH 5, at a ratio of 10:1 (buffer/dry substrate), under agitation of 150 rpm for 60 min at room temperature. Solids were separated by filtration through a nylon cloth followed by centrifugation at 15000 g for 10 minutes, and the clarified supernatants were frozen and stored for subsequent enzymatic analysis. Experiments were carried out with three replicates for each medium composition and each incubation time.

### Enzymatic assays

All enzymatic assays were carried out in sodium acetate buffer, 100 mM, pH 5, at 50 °C in triplicate and the mean values calculated. Relative standard deviations of measurements were below 5%. FPase and endoglucanase activities were determined using Whatman No. 1 filter paper and carboxymethilcellulose as substrates respectively^43^. The total reducing sugar liberated during the enzymatic assays were quantified by the dinitrosalicylic acid (DNS) method^44^ using glucose as a standard.

Xylanase and mannanase activities were determined in beechwood xylan (final concentration of 1% w/v) and locust bean gum (0.4% w/v), respectively. The enzymatic reactions were initiated with addition of 100 mL of enzyme extract diluted to 400 μL substrate solution with the polysaccharide prepared in buffer. The reaction mixtures were incubated for 30 min and the amount of reducing sugars released determined by the DNS method using xylose and galacturonic acid as standards. Activities β-glucosidase, β-xylosidase, α-galactosidase and arabinofuranosidase were measured using ρPNβ-Glc, ρNPβ-Xyl, ρNPα-Gal and ρNPα-Ara as substrates, respectively. The reaction mixtures contained 30 μL enzyme solution was diluted 50 μL synthetic substrate solution (1 uM final concentration) and 20 buffer μL. The reaction mixture was incubated for 15 min and quenched with 100 μL of a sodium carbonate solution (0.5 M). Absorbance was measured at 410 nm and the amount of ρ-nitrophenol released assessed by a standard curve. One enzyme activity unit (U) was defined as the amount of enzyme which released a μmol of the product (equivalent glucose, xylose and ρ-nitrophenol) per minute under assay conditions used for all activities.

### Protein determination

Protein concentration in the enzymatic extracts was determined by the Coomassie Blue binding method using bovine serum albumin as the standard^45^.

### Biomass saccharification

The crude enzymatic extracts produced by *C*. *cubensis* and com- mercial cellulase (Multifect® CL) were applied in a biomass saccharification experiment. The *C*. *cubensis* enzymatic extract were concentrated 5-fold before the experiment using an Amicon Ultra- filtration system (Millipore Co. – Billerica, MA, USA) and an YM-10 (Cut-off Mr 10,000 Da) membrane filter. Enzymatic saccharification of alkali-treated sugarcane bagasse was performed in 2 mL sample tubes at an initial solid concentration of 2% dry matter (w/v) in 1.5 mL of 50 mM sodium acetate buffer at pH 4.5. Enzyme loading was specified as 10 FPase units per gram of biomass with the addition of sodium azide (10 mM) and tetracycline (40 μg mL^−1^) to the reaction mixture to inhibit microbial contamination. The reaction was carried out in an orbital shaker at 250 rpm and 50 °C for different time intervals up to 72 h. These samples were immediately heated to 100 °C to denature the enzymes, cooled and then centrifuged for 5 min at 15,000 g. Products of the saccharification assays were analyzed by high performance liquid chromatography (HPLC) with a Shimadzu series 10 A chromatograph. The HPLC was equipped with an Aminex HPX-87P column (300 × 7.8 mm) and refractive index detectors. The column was eluted with water at a flow rate of 0.6 mL min^−1^ and 80 °C.

### Statistical Analysis of Data

The values of xylanase activities on different substrates were analyzed using Assistat 7.7 software, performing analysis of variance (ANOVA) followed by Tukey’s test at a significance level of 5% (*α* = 0.05). The standard deviation was also calculated for all assays.
